# Butterflies embrace maladaptation and raise fitness in colonizing novel host

**DOI:** 10.1111/eva.12775

**Published:** 2019-02-26

**Authors:** Michael C. Singer, Camille Parmesan

**Affiliations:** ^1^ Theoretical and Experimental Ecology Station UMR 5321 CNRS and Paul Sabatier University Moulis France; ^2^ Biological and Marine Sciences, Portland Square Building University of Plymouth Plymouth UK; ^3^ Geological Sciences University of Texas at Austin Austin Texas

**Keywords:** adaptation, adaptive landscape, butterfly, ecological fitting, *Euphydryas*, host shift, land‐use change, maladaptation, novel host

## Abstract

We illustrate an evolutionary host shift driven by increased fitness on a novel host, despite maladaptation to it in six separate host‐adaptive traits. Here, local adaptation is defined as possession of traits that provide advantage in specific environmental contexts; thus individuals can have higher fitness in benign environments to which they are maladapted than in demanding environments to which they are well adapted. A population of the butterfly *Euphydryas editha *adapted to a long‐lived, chemically well‐defended host, *Pedicularis,* had traditionally been under natural selection to avoid the ephemeral, less‐defended *Collinsia*. The lifespan of *Collinsia* was so short that it senesced before larvae entered diapause. After logging killed *Pedicularis *in clear‐cut patches and controlled burning simultaneously extended *Collinsia* lifespan, insect fitness on *Collinsia* in clearings suddenly became higher than on *Pedicularis* in adjacent unlogged patches. *Collinsia *was rapidly colonized and preference for it evolved, but insects feeding on it retained adaptations to *Pedicularis* in alighting bias, two aspects of postalighting oviposition preference, dispersal bias, geotaxis, and clutch size, all acting as maladaptations to *Collinsia*. Nonetheless, populations boomed on *Collinsia* in clearings, creating sources that fed pseudosinks in unlogged patches where *Pedicularis* was still used. After c. 20 years, butterfly populations in clearings disappeared and the metapopulation reverted to *Pedicularis*‐feeding. Here we show, via experimental manipulation of oviposition by local *Pedicularis*‐adapted and imported *Collinsia*‐adapted butterflies, that the highest survival at that time would have been from eggs laid in clearings by butterflies adapted to *Collinsia*. Second highest were locals on *Pedicularis*. In third place would have been locals on *Collinsia *in clearings, because local females maladaptively preferred senescent plants. *Collinsia* had been colonized despite maladaptation and, after successional changes, abandoned because of it. However, the abandoned *Collinsia* could still have provided the highest fitness, given appropriate adaptation. The butterflies had tumbled down an adaptive peak.

## 1 INTRODUCTION

### 1.1 Maladaptation: How to define and identify it?

In this paper, we are not concerned with adaptation and maladaptation as processes, but as conditions, both of the whole organism and of its traits that affect its fate. Adaptation in this sense, as a state of being, is never perfect, so at what point along its continuous distribution does it become “mal?” Fisher (1930) wrote: “an organism is regarded as adapted to a particular situation only in so far as we can imagine…slightly different…forms, which would be less well adapted to that environment.” Here, we invert this statement and regard an organism as maladapted to its environment when we cannot merely imagine, but discover and study, different forms of the same species which exist and which are demonstrably better‐adapted to that environment.

Kawecki and Ebert (2004) define “local adaptation” as “possession of traits that provide an advantage under local environmental conditions, regardless of the consequences of those traits for fitness in other habitats.” We adopt this concept. By doing so we can describe an individual as maladapted to a habitat in which its traits fail to maximize fitness, even if its fitness is higher in that habitat than it would be in a different habitat where the same traits did maximize fitness. A group of such individuals could form a population that would attain higher absolute fitness in a benign environment to which the population is maladapted than in a demanding environment to which it is well adapted. Individuals that were biased to emigrate from habitats to which they were maladapted into patches to which they were adapted would thereby lower their fitness. Habitat patches acting as sources would be the patches to which the organisms were maladapted. This use of terminology may seem perversely paradoxical, but it allows us to describe specific events that we have observed and studied, without tying ourselves in any further verbal knots that we just did.

Brady et al., (in review) note that individuals or populations have been described as “maladapted” in reference to their performance, their fitness, or their traits that are (sometimes wrongly) assumed to be surrogates for fitness. Here, partly in review of our group's prior work and partly from new data, we use a diversity of metrics, including population growth rates, individual survival, and known host‐adaptive traits, to address adaptation/maladaptation of a metapopulation of Edith's checkerspot butterfly (*Euphydryas editha*) to its novel and traditional hosts during a bout of rapid anthropogenic diet evolution that began around 1967 at Rabbit Meadow, Tulare Co., California. In describing this long‐term evolutionary study, we will demonstrate all of the unexpected and apparently paradoxical roles of maladaptation that we list above. We also give a brief comparison with a recently published, entirely independent host shift at Schneider's Meadow, Carson City, Nevada, by the same butterfly species. Just as at Rabbit Meadow, the shift at Schneider was driven by higher fitness on the novel than on the traditional host, though the reasons for this fitness effect were not the same at the two study sites.

Below, we describe, in the order in which they occur, the sequence of behavioral traits that a female *E. editha* manifests as she approaches a plant, assesses it for oviposition, and handles it once a positive decision has been made. We begin by assessing the roles of these traits in adaptation and maladaptation to novel and traditional hosts at the Rabbit Meadow metapopulation during a time period when both hosts were used. Summarizing largely published results, we show that anthropogenic disturbance rendered fitness consistently higher on the novel host despite maladaptation to it in six separate traits.

After about 20 years, populations in all of the colonized patches went extinct. Here we ask whether there was a role of persistent maladaptation to the novel host in driving those extinctions. To address this question, we use a previously unpublished experiment that demonstrates reversal of the fitness relationship between insects using traditional and novel hosts. The experiment not only documents restoration of higher fitness on the traditional host, but shows the role of maladaptation to the novel host in driving its final abandonment. In adopting a narrative style, we strive to render a complex story digestible.

## 2 STUDY SPECIES: EDITH'S CHECKERSPOT AND ITS HOSTS

### 2.1 Distribution, life history, and life‐history trade‐offs

Edith's checkerspot (*Euphydryas editha)* is a sedentary (Ehrlich, 1961; Harrison, 1989) thermophilic (Weiss, Murphy, & White, 1988) Nymphaline butterfly distributed in scattered, mostly isolated, populations, and metapopulations across Western North America from Baja California in the south to Central Alberta at its poleward limit, and from sea level to around 3,600 m elevation (Ehrlich & Hanski, 2004). Any one of five or six host genera in the Plantaginaceae or Orobanchaceae may serve as the principal host of an *E. editha* population. In a sample of 57 populations, 43 were monophagous (despite many having multiple potential host species available), with the remainder using two to four host genera (Singer & Wee, 2005).

The butterfly is univoltine, that is, restricted to one generation per year. In most habitats, except at the highest elevations, active life stages are confined to spring and early summer and the insects spend the rest of the year diapausing about halfway through their larval stage. Eggs take about two weeks to hatch and young larvae must feed for another two weeks before reaching a size at which they can diapause; therefore, a plant chosen by an ovipositing butterfly must remain edible for a month if it is to support offspring to diapause. At the elevation of our study sites (c. 2,300 m), diapause lasts about 9 months through most of summer, all of autumn and winter. It is broken at snowmelt, after which postdiapause larvae develop rapidly. When a female postdiapause larva has achieved a size at which she has the potential to pupate, which usually occurs in late April or May at 2,300 m, any additional time spent feeding increases fecundity but delays oviposition. If the host is an ephemeral annual *Collinsia,* delayed oviposition increases offspring mortality from host senescence, which usually occurs in late June or July. A trade‐off is created between maternal fecundity and offspring mortality (Singer & Parmesan, 2010).

The typical response of checkerspot populations to this fecundity/mortality trade‐off has been twofold: (a) to mitigate the trade‐off by evolving oviposition preference for individual host plants that are not yet senescent (Singer & McBride, 2010) and (b) to evolve a life history in which fecundity is high and oviposition is thereby delayed, rendering the insect life cycle asynchronous with that of its host. In consequence of the asynchrony, high larval mortality caused by host senescence is routine (Singer, 1972; Singer & Parmesan, 2010).

### 2.2 Baseline for changes: Adaptive host use in traditional, stable environments

*Euphydryas editha* frequently chooses to oviposit on different host species at different sites despite the presence of almost identical plant communities. Reciprocal transplant experiments have shown that this geographical variation of host use was mechanistically driven both by heritable variation of oviposition preference among insect populations and by heritable variation of acceptability among plant populations (Singer & McBride, 2012; Singer & Parmesan, 1993). Variation of host use within sites was driven by interactions between strength of preference and encounter rates with different potential hosts, in addition to heritable variation of oviposition preference (Singer, 1983, Singer, Ng, & Thomas, 1988; Singer, Vasco, Parmesan, Thomas, & Ng, 1992; see Section 10 for definitions of host use, insect preference, strength of preference, and host acceptability).

**Figure 1:**
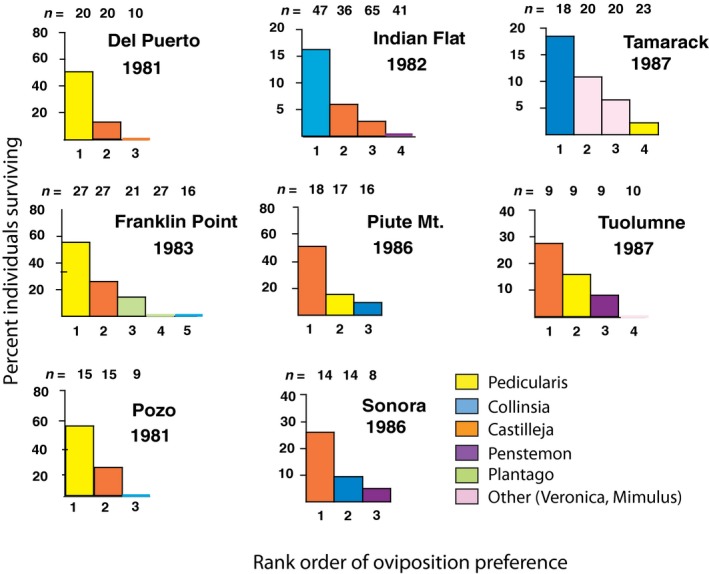
Adaptive variation of oviposition preferences across populations where diet was not currently evolving (previously published; Figure modified from Singer et al., 1994). For each population, hosts used and potential hosts available (species not used at the site but serving as principal hosts elsewhere) are arranged from left to right in the order of adult oviposition preferences as determined from behavioral tests. The height of the bars shows survival of early stages from manipulated ovipositions or from placing out neonate larvae, all carried out in the field (wild butterflies on naturally growing plants in the population). The fact the bars “step down” from left to right shows that, within each site, the oviposition preference hierarchy was concordant with the rank order of hosts in supporting offspring survival. Transplants among sites have also been done (Singer & McBride, 2012; Singer & Parmesan, 1993), but this Figure carries no information about them. For example, while the Figure shows that *Collinsia* was preferred at Tamarack Ridge and *Pedicularis* at Pozo, it does not tell us which host species Tamarack butterflies would prefer or how their larvae would survive if they were tested at Pozo. The Figure does not show *Collinsia* as a host at Del Puerto Canyon because it was not available (i.e., dead) at the season when eggs were laid in the year the experiment was done; however, in one prior year (1983) it had been available and used by the butterflies

In prior work, we classified plant species that are known to serve as the sole or principal hosts of *E. editha* as “potential hosts” at sites where they are not used. We then conducted experiments in which neonate larvae were placed on actual and potential hosts in a set of eight *E. editha* populations that were not currently engaged in host shifts. Within each population, the rank order of hosts and potential hosts in the oviposition preference hierarchy was concordant with the rank order of the same plants in their support of offspring survival (Figure 1, modified from Singer, Thomas, Billington, & Parmesan, 1994). This concordance stemmed at least partly from local adaptation by each population to its traditional host (Singer & McBride, 2012).

Despite extensive ecotypic variation, and despite hybrids between populations adapted to different hosts suffering reduced fitness on both parental hosts (McBride & Singer, 2010), genetic analysis of 40 *E. editha* populations showed no trend for isolation by host. Isolation by distance was, in contrast, strong and consistent. These populations did not comprise a set of host‐associated cryptic species (Mikheyev et al., 2013).

### 2.3 Host choice behavior and host‐adaptive traits

Unlike most flying insects, for which olfaction is important in host location (Bruce, Wadhams, & Woodcock, 2005), *E. editha* find their hosts visually (Parmesan, 1991; Parmesan, Singer, & Harris, 1995). A positive response by a female to visual stimuli is to alight on a plant and taste it with her atrophied foretarsi. A positive response to plant chemistry, definitely including taste (Singer & McBride, 2010) but not (yet) shown to include olfaction, is to choose a height above the ground at which to search with the ovipositor for tactile stimuli. A positive response to tactile stimuli is, finally, to lay a clutch of eggs. In some populations, one clutch per day is laid, containing all the eggs that are mature within the female when the clutch is laid; in other populations, clutches are smaller and several can be laid in quick succession (McBride & Singer, 2010; Singer & McBride, 2010).

The height above the ground at which eggs are laid strongly affects both susceptibility to incidental predation by grazers and exposure to thermal stress (Bennett, Severns, Parmesan, & Singer, 2015). Where eggs are laid low, the behavioral mechanism that achieves this is geotaxis. A positively geotactic female drops to the ground after accepting plant taste, curling her abdomen into a ¾ circle, extruding her ovipositor and probing with it to seek the base of the host. This video https://doi.org/10.1371/journal.pbio.1000529.s015 shows the geotactic behavior: a female is placed on a *Pedicularis* plant in her natural habitat. Initially, she basks. Then, after being gently reminded by the experimenter (Lindy McBride) that she has a task to perform, she tastes a leaf, and as a positive response to taste, searches for the base of the plant. Failing to find it, she takes off, re‐alights naturally on the same leaf of the same plant, tastes it again, and tries again to find the base of the plant, this time successfully.

In populations where eggs are laid further from the ground, females are nongeotactic and usually oviposit close to the point of alighting. This video shows nongeotactic oviposition on *Collinsia* preceded by the tasting behavior, with the insect tapping the plant by extending her foretarsi: https://www.youtube.com/watch?v=pXT4qinQ0KM Both videos illustrate the manipulability of the butterflies, which behave naturally after being placed by hand on potential hosts, thereby facilitating the testing of their postalighting oviposition preferences.

### 2.4 Study ecotypes

The Rabbit Meadow metapopulation of *E. editha* on which we focus here comprises one member of a series that occurs along the western slopes of the Sierra Nevada in California at 2,000–2,800 m elevation. In some of these metapopulations, the butterflies oviposit on ephemeral annual *Collinsias,* while in others they choose persistent perennials in the genera *Pedicularis* and/or *Castilleja*. The two sets of metapopulations are distributed in a geographical mosaic, with insects in each set adapted to the host(s) that they use in a suite of behavioral and developmental traits (Singer & McBride, 2010).

*Collinsia* was the most abundant host at all sites and was no less abundant at sites where the butterflies failed to choose it, so the local choice of *Collinsia* or *Pedicularis *was unrelated to host abundance. Instead, it was driven evolutionarily by inter‐site variation of *Collinsia *lifespan and mechanistically by inter‐site variation of butterfly oviposition preference (Singer & McBride, 2012). *Collinsia* was used for oviposition by the butterflies at sites where it was most long‐lived. At sites where *Collinsia* lifespan was shortest, *Collinsias* were blooming and available for oviposition when the butterflies were flying. However, very few individual plants lived long enough to nourish young larvae to diapause, causing natural selection against oviposition on them (Singer & McBride, 2012). At these sites, the butterflies preferred to oviposit on *Pedicularis* and had evolved the appropriate suite of adaptations to use it (Singer & McBride, 2010). This was the situation at the Rabbit Meadow metapopulation prior to the anthropogenic host shift to *Collinsia* that we will describe.

### 2.5 *Pedicularis* as a “demanding” host

In our discussion of a host shift from *Pedicularis* to *Collinsia* and back again, we will assume that, where both hosts are phenologically available, *Pedicularis* is the better‐defended of the two. This section summarizes the evidence for that assumption, which comes from experiments in which growth rates and survival of neonate larvae were measured after the larvae had been experimentally placed on cut stems of the two hosts under shade cloth in a natural habitat. The experimental *Collinsia* plants were not senescent, so mortality that naturally occurs from host senescence was not included in the experiment. The result was that *E. edith*a larvae from Sierra Nevada populations adapted to *Pedicularis* survived well on *Collinsia*, while larvae from populations adapted to *Collinsia *suffered extremely high mortality on *Pedicularis *(Singer & McBride, 2010).

Further, experimentally fed larvae from two distantly related *Pedicularis*‐adapted metapopulations grew faster and weighed more at ten days of age on *Collinsia* than on their own host, *Pedicularis* (Singer & McBride, 2010, their Table 5). However, larvae from two *Collinsia*‐adapted metapopulations grew even faster on *Collinsia* than those from the two *Pedicularis*‐adapted metapopulations (Singer & McBride, 2010). So, in sum, in manipulative trials where individual *Collinsia* plants were chosen by experimenters to be blooming rather than senescent, the fastest growth was of *Collinsia*‐adapted larvae on *Collinsia*, second fastest were *Pedicularis*‐adapted larvae on *Collinsia,* and third were *Pedicularis*‐adapted larvae on *Pedicularis*.

These results strongly suggest that *Pedicularis* forms a more demanding nutritional environment than *Collinsia. *In presumed response to this asymmetry, most butterflies from *Pedicularis*‐adapted populations will oviposit on *Collinsia,* especially if they fail to find *Pedicularis* quickly, while most butterflies from *Collinsia*‐adapted populations reject *Pedicularis,* even after failing to find their own host for more than a day (Singer & McBride, 2010).

## 3 RABBIT MEADOW HOST SHIFT

### 3.1 Initial phase: survival and population growth higher on novel than on traditional host

**Figure 2:**
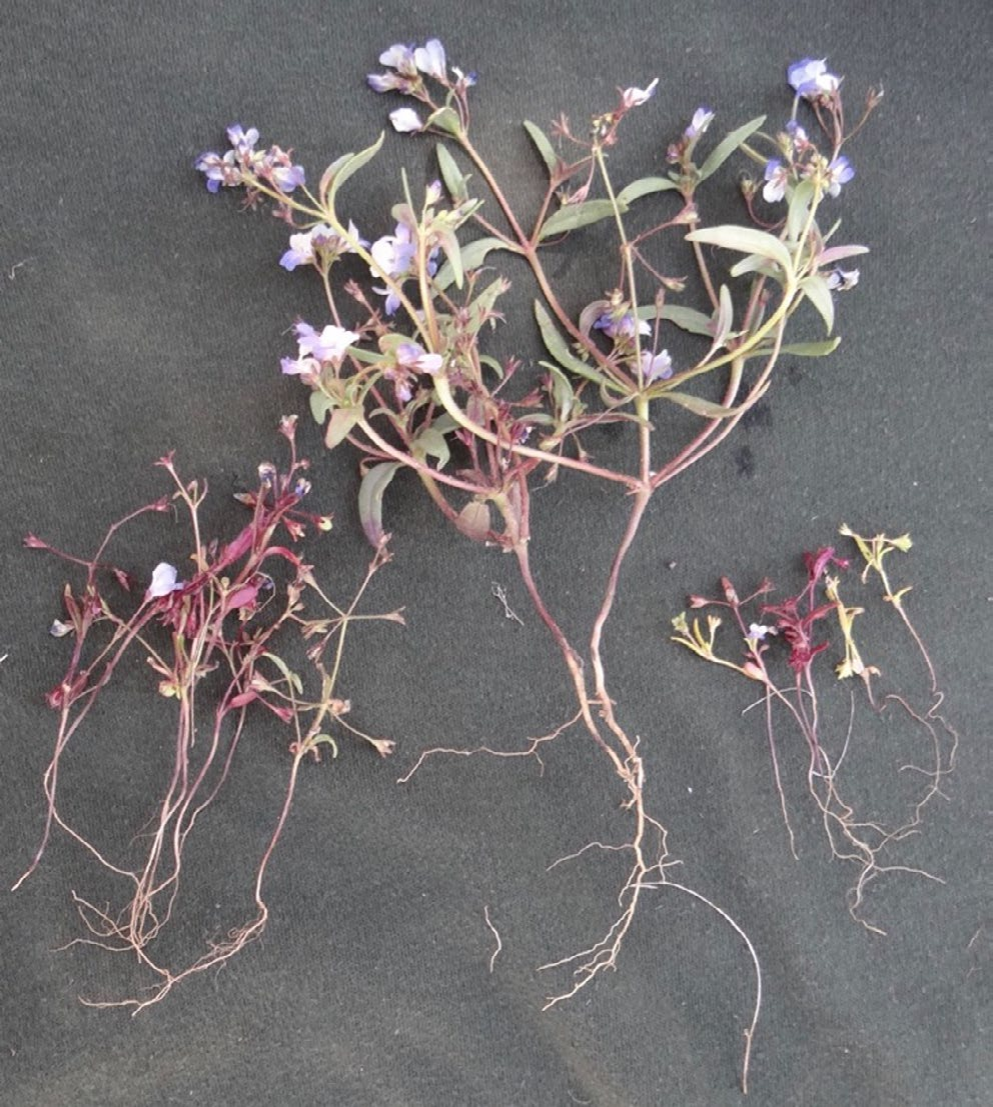
Persistent effects on size and longevity of *Collinsia torreyi* at Rabbit Meadow of a fire set by loggers between 1967 and 1977. All plants were gathered on the same day: June 15, 2018. On the right, typical individuals from an unlogged patch. All are senescent but still edible to *E. editha* larvae. The variation of color (reds and yellows) reflects chemical polymorphism, not variable phenology. In the center and at left are individuals from a clearing, found growing within a meter of each other and illustrating the variable phenology that allowed butterflies to choose budding, blooming or senescent plants in 2002–2003. Plants at left would be classed as senescent, except for one blooming individual. Plants in the center would be classed as “budding” since they bear unopened flower buds

**Figure 3:**
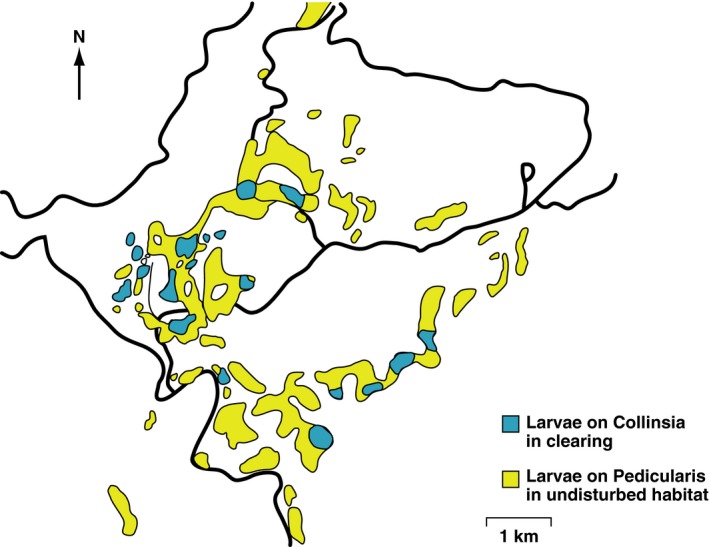
Distribution of *E. editha* oviposition, deduced from prediapause larval webs, across logged and unlogged patches at the Rabbit metapopulation in 1986 (previously published; figure modified from Thomas et al., 1996)

In the ancestral condition at Rabbit Meadow, oviposition was principally on *Pedicularis semibarbata* with minor use of a rarer host, *Castilleja disticha* (Singer, 1983; Singer & Thomas, 1996). When our work started in 1979, we discovered that the U.S. Forest Service had inadvertently set up an evolutionary experiment that would otherwise have been difficult and expensive to organize. Starting around 1967 and continuing through the 1970s, loggers had created a set of interdigitating patches of two distinct habitat types, clearings and unlogged patches, distributed across 8 × 10 km. In the cleared patches, *Pedicularis* had been killed by removal of trees (it is a hemiparasite of gymnosperms) and the lifespan of *Collinsia torreyi* had been extended by the fertilizing effect of postlogging fires (Figure 2). As a result, the time constraint for development on *Collinsia* had been released and natural selection suddenly favored oviposition on *Collinsia *in clearings, while continuing to strongly oppose this host choice in adjacent unlogged patches (Singer, 2015; Singer & Thomas, 1996). By 1979, the butterflies had begun to colonize *Collinsia* in the clearings, and by the mid‐1980s, the larger clearings had all been colonized (Figure 3). The scale of the habitat mosaic permitted the insects to move among patches and express their preferences for both patch type and host species (Singer, 2015; Thomas & Singer, 1987).

For three years, from 1984 to 1986 Moore (1989) followed the fates of naturally laid *E. editha* egg clusters in both habitat types at Rabbit Meadow and obtained direct estimates of survival in nature through each life‐history stage. In both 1985 and 1986, survival was higher on *Collinsia *in a clearing than on *Pedicularis* in an adjacent unlogged patch; in 1984, there was little difference between survival on the two hosts. More generally across the metapopulation in the 1980s, both year‐to‐year population growth rates and adult density were higher in clearings than in unlogged patches (Thomas, Singer, & Boughton, 1996). Mark–release–recapture experiments showed that clearing populations acted as sources while populations in unlogged patches acted as pseudosinks, absorbing emigrants from clearings and suffering increased intraspecific competition as a result (Boughton, 1999; for definition of “pseudosink,” see Section 10 and Watkinson & Sutherland, 1995). High survival on the novel host was not due to release from parasitoid attack, since there was no such release (Moore, 1989).

### 3.2 Middle and terminal phases: alternative stable states and abandonment of novel host

During the 1990s, the system oscillated between two stable states, one of which was the source‐pseudosink system just described. In the alternate state, first triggered when an unseasonal frost in 1992 temporarily extinguished the clearing populations, the unlogged patches acted as sources and the clearings became true sinks with no survival of larvae to diapause (Boughton, 1999, cf Ronce & Kirkpatrick, 2001). In 2001–2002, the oscillations stopped. By June 2002, the clearings and their *Collinsia* host had been abandoned, and so they have remained to the present day (June 2018).

Since our theme is adaptation and maladaptation, not metapopulation dynamics, we will gloss over the complexity of the transitional phases in the 1990s and discuss only two time periods:

1. Initial phase of *Collinsia* use: 1981–1988, soon after colonization of logged clearings that were not available prior to 1967.
2. Terminal phase of *Collinsia* use: years 2002–2003, immediately after *Collinsia* had been abandoned.

Below, we document the roles played by maladaptation to *Collinsia* in these two time periods. We made use of the availability of insect populations representing both the starting‐points and evolutionary targets of the host shift. The starting condition was represented by a *Pedicularis*‐adapted metapopulation of *E. editha* at a site unaffected by logging, 12 km to the south of Rabbit Meadow at Colony Meadow in Sequoia National Park. The evolutionary target was represented by *Collinsia*‐feeding *E. editha* at Tamarack Ridge 60 km to the north of Rabbit Meadow, a metapopulation that showed the full suite of adaptations to *Collinsia *and thus represented the unfulfilled target of the Rabbit Meadow host shift (Singer & McBride, 2010).

## 4 MALADAPTATION TO NOVEL HOST: HOST‐ADAPTIVE TRAITS

Experiments have shown how colonization of a novel resource can incur initial multi‐trait maladaptation that generates rapid evolutionary response if sufficient genetic variation exists and high risk of population extinction if it does not (Agashe, Falk, & Bolnick, 2011). In our own observations, we document initial multi‐trait maladaptations that led to rapid evolutionary adaptation in at least one host‐adaptive trait. We will show how the apparent failure of a different trait to adapt, combined with environmental change, led to eventual population extinction on the novel resource.

Below, we list the maladaptations to *Collinsia* that resulted from retention of adaptations to *Pedicularis* by *Collinsia*‐feeding *E. editha* in the clearings at Rabbit Meadow during the period when *Collinsia* acted as their novel host in the 1980s. The order of traits in the list is the order in which these behaviors occur during an oviposition search.

### 4.1 Maladaptive traits and their effects on fitness

#### 4.1.1 First maladaptation: alighting bias—inefficient search for *Collinsia*

**Figure 4:**
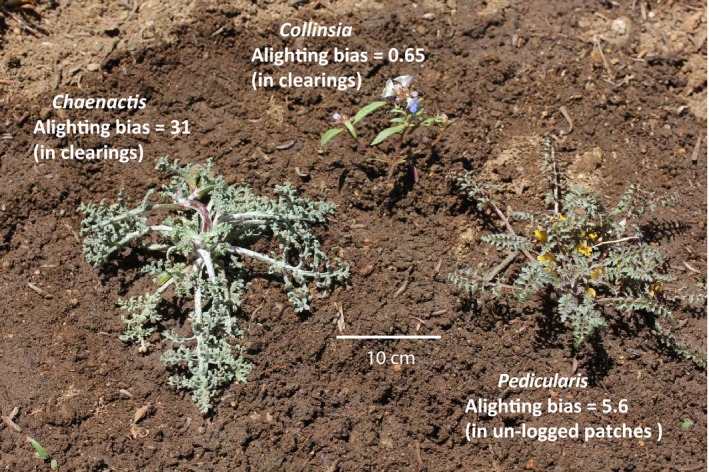
Hosts and a nonhost at Rabbit Meadow, illustrating visual resemblance between the nonhost *Chaenactis douglasii* (Asteraceae) and the host *Pedicularis semibarbata* (Orobanchaceae) (similar photo in Singer, 2015). Alighting biases are the proportions of actual alights on host plants relative to those expected from randomly alighting on vegetation (Parmesan et al., 1995). A value lower than 1 indicates avoidance of the plant

Wild butterflies observed in natural oviposition search in an unlogged patch of the Rabbit Meadow metapopulation found *Pedicularis* efficiently but those searching in an adjacent clearing found *Collinsia* inefficiently; either randomly (Mackay, 1985) or significantly less often than they would have done in random search (Parmesan et al., 1995). In the clearing habitat, Parmesan et al. (1995) found strong positive alighting biases toward nonhosts that visually resembled the absent *Pediculari*s, especially toward *Chaenactis douglasii* (Asteraceae; Figure 4). However, *Chaenactis *received no eggs: the butterflies repeatedly alighted on it, tasted it, and moved on. Figure 4 shows, in the body of the Figure, the alighting biases toward both hosts and *Chaenactis.*

Like all behaviors involved in host choice by *E. editha* (McNeely & Singer, 2001), the alighting bias toward *Pedicularis* was not learned (Parmesan et al., 1995). When naïve mated females originating from the clearing were held captive until strongly motivated to oviposit and then experimentally released in the adjacent unlogged patch, their bias toward *Pedicularis* was just as strong in the very first alights of their very first searches as it was in alights by experienced butterflies naturally flying across the same habitat patch (Parmesan et al., 1995).

Because *Collinsia* was abundant (14% of vegetation), the butterflies’ rate of encounter with this host was high, despite inefficiency of search. Naïve searching females in a clearing alighted on *Collinsia* on average once every 7.7 min, while those in a neighboring unlogged patch alighted on *Pedicularis *(8% of vegetation) once every 2.6 min (Parmesan et al., 1995).

However, the combination of postalighting preference for *Pedicularis* and difficulty of finding acceptable physical oviposition sites on *Collinsia* (see section 4.1.5) reduced the rate of actual oviposition on *Collinsia *below that expected from the rate at which it was encountered. Parmesan et al. (1995) gathered naïve females emerging from *Collinsia* in a clearing, waited for them to be sufficiently motivated that they would attempt to oviposit on either host after alighting, released them, and observed their oviposition searches. 89% of those released in an unlogged patch (*n* = 27) succeeded in laying eggs on *Pedicularis* after searches averaging 11 min. In contrast, a mere 20% of those released among *Collinsias* in the clearing (*n* = 40) succeeded in ovipositing, despite longer searches averaging 29 min.

Parmesan et al.’s (1995) observations of inefficient (i.e., worse than random) search for *Collinsia torreyi* raise the question of whether efficient search for these small, potentially unapparent (see Section 10), plants can be achieved by *E. editha*. Yes, it can. In a population (Schneider's Meadow) adapted to a visually similar species of *Collinsia, C. parviflora, *the butterflies did search efficiently for their host. *Collinsia *comprised 12% of the vegetation and received 71% of alightings (Parmesan, 1991). Thus, the bias toward *Collinsia* at Schneider's Meadow, where it was the traditional host, was equivalent to the bias toward *Pedicularis* at Rabbit Meadow, both being alighted on by local females about six times more than expected at random. Therefore, the inefficiency of *Collinsia* search by butterflies in the Rabbit Meadow clearings, where it was a novel host, was ascribed by Parmesan (1991) to evolutionary lag, not to constraint. We consider it unlikely that gene flow from unlogged patches played a major constraining role because clearings were acting as sources when the search‐behavior study was done and postalighting oviposition preference for *Collinsia* was already evolving in clearings in response to patch‐specific natural selection (see below).

#### 4.1.2 Second maladaptation: reduction of realized fecundity caused by postalighting host preference for *Pedicularis*

Since *Pedicularis* had been killed in the clearings, butterflies emerging there that preferred *Pedicularis* were forced to either emigrate or undertake prolonged search until they reached a level of oviposition motivation at which they would accept *Collinsia* (Singer, Vasco et al., 1992).

In 1983, we estimated the average consequence for fecundity of emigrating or remaining in the clearing. We captured teneral (newly emerged) butterflies emerging from *Collinsia* in a clearing and experimentally exposed them to either *Pedicularis* or *Collinsia* for 5 days. The reduction in mean fecundity from forcing the butterflies to use *Collinsia* was 30%, from 171 eggs in 5 days among butterflies exposed only to *Pedicularis* down to 120 in those offered only *Collinsia *(Singer, 2015).

#### 4.1.3 Third maladaptation: postalighting preference for senescent over blooming *Collinsia*

The ability to discriminate among phenologically differing host individuals is an important axis of evolution in herbivorous insects (Janz & Nylin, 1997). Prior behavioral preference tests with *E. editha* showed that, when offered *Collinsia *plants chosen by the experimenter to have contrasting phenological states, butterflies from the metapopulation at Tamarack Ridge preferred blooming over senescent plants, as expected in a population adapted to *Collinsia*. In contrast, Rabbit Meadow insects maladaptively preferred senescent over blooming plants (Singer & McBride, 2010).

Here we present data from unpublished censuses to ask whether these experimentally tested preferences for host phenology resulted in the expected distributions of young (first and second instar) larvae across phenologically differing *Collinsias* in nature. Working in a Rabbit Meadow clearing in 1986, we used random numbers to place out 24 quadrats, each measuring 30 cm × 30 cm. Although the quadrats were not selected to contain *Collinsia*, they all did so. Ten of them also contained groups of *E. editha* larvae in their conspicuous communal webs. Within each of those ten quadrats, we classified individual plants as phenological condition 1 = budding (still bearing at least some unopened buds), 2 = blooming (open flowers but no buds), 3 = edible senescent (leaves and bracts edible but no remaining flowers or buds), or 4 = dead (really dead). Using these numbers, we calculated two statistics for each quadrat: the mean phenological state of all the *Collinsia* plants in the quadrat and the mean phenological state of the plants that bore eggs or larvae. In 1991, we made similar measures at Tamarack Ridge.

**Figure 5:**
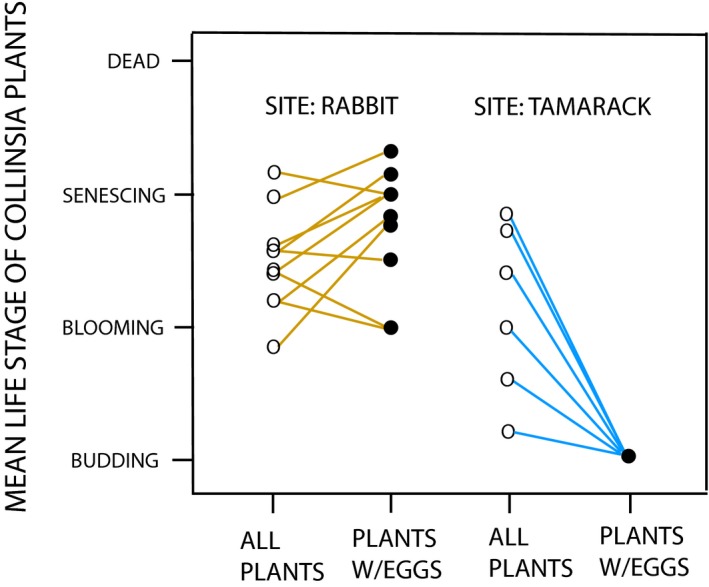
Mean phenologies of all *Collinsia* plants within random 30 cm quadrats (open circles) and mean phenologies in the same quadrats of all *Collinsia* plants naturally chosen for oviposition by wild, free‐flying females and bearing natural eggs or larval webs (closed circles). Left hand panel: Rabbit Meadow metapopulation; right hand panel Tamarack Ridge metapopulation (all data previously unpublished). We ranked the order of slopes in the two combined panels, starting from the strongest bias toward laying eggs on budding plants (within a quadrat) and ending with the strongest bias toward senescent plants. With Tamarack Ridge represented by T and Rabbit Meadow by R, the order of ranks, as can be seen on the Figure, is TTTTTRRTRRRRRRR. Using these ranks, a Mann–Whitney U test, two‐tailed, gives the significance of the difference between the two sites as *p* = 0.004. The distribution of larvae at Tamarack Ridge was significantly more biased toward young *Collinsia *plants than the equivalent distribution at Rabbit Meadow

The relationship between mean phenological state of plants with *E. editha* and that of all plants in the same quadrat differed significantly between the two metapopulations in the direction expected from the behavioral trials (Figure 5). The distribution of larvae at Rabbit Meadow was significantly less biased toward young plants than at Tamarack Ridge, with resulting higher mortality at Rabbit. This was first quantified by Moore (1989), who estimated the percentages of natural egg clutches in a Rabbit Meadow clearing that failed, either as eggs or as larvae, because the host died before larvae were sufficiently mature to diapause. These mortality estimates were 16% in 1984, 11% in 1985, and 6% in 1986.

#### 4.1.4 Fourth maladaptation: biased dispersal away from the clearing habitats that provided higher fitness; failure to colonize small, high‐quality patches of novel host

This section is complex, but readers can accept the header at face value and skip the details below without loss of continuity. Like most butterflies (Friberg, Olofsson, Berger, Karlsson, & Wiklund, 2008; Wiklund, 1977), *E. editha* possess separate but related preferences for hosts and habitats. Individuals enter and assess habitat patches based principally on their physical characteristics, such as openness and slope aspect. Only after a female has entered a patch can she discover its quality in terms of nectar sources, roosting sites, and oviposition sites. The butterflies at Rabbit Meadow were presented with two patch types: clearings and unlogged patches. During the early phase of clearing colonization, there was net dispersal out of the clearings, where densities were higher. However, dispersal was not density‐dependent (Boughton, 2000), so the tendency to leave clearings was an expression of habitat preference rather than a response to density (Boughton, 1999, 2000; Singer, 2015; Thomas et al., 1996).

The match or mismatch between her postalighting oviposition preference and the identities of the potential hosts that she encounters is only one aspect of habitat quality that a female uses to decide whether to stay or leave (Singer, 2015). But use it she does (Hanski & Singer, 2001), with the result that the reciprocal movement of *E. editha* between clearing and unlogged patches was associated with their individual host preferences (Thomas & Singer, 1987). As expected (Bolnick et al., 2009; Clobert, Gaillard, Cote, Meylan, & Massot, 2009; Edelaar & Bolnick, 2012), this biased dispersal generated an adaptive difference between the two habitat types in postalighting preferences. Butterflies in the recently colonized clearings were more accepting of *Collinsia* after alighting than those in adjacent unlogged patches. The difference between the patch types in postalighting preference was heritable and generated more by biased dispersal than by patch‐specific natural selection (Singer & Thomas, 1996).

However, by dispersing out of the clearings into unlogged patches and choosing the habitats to which they were best‐adapted, most of the butterflies reduced their own fitnesses. There were a few females, those that were most strongly *Pedicularis*‐preferring, that would have delayed oviposition for several days if they remained in their natal clearing habitats (Singer, 2015; see below). Because these females were a minority, the mean penalty in realized fecundity for staying in the clearings (above) was insufficient to outweigh the survival penalty incurred by moving to the more demanding host in the unlogged habitat (Singer, 2015). Thus, most females that emerged in the clearings gained an overall fitness benefit if they remained in the clearings for oviposition.

Although migrants from clearings into unlogged patches were biased to be more *Pedicularis*‐preferring than their fellows that remained in the clearings, they were LESS *Pedicularis*‐preferring and more *Collinsia*‐accepting than the mean preferences in the unlogged patches where they arrived. Therefore, in addition to increasing intraspecific competition in the patches that received them (Boughton, 1999, 2000), they drove evolution of postalighting oviposition preference in *Pedicularis *patches in a locally maladaptive direction.

Evidence for these twin effects on population dynamics and adaptation comes from measurements taken in unlogged patches at Rabbit Meadow during the 1980s. Two separate metrics, population density and *Collinsia* acceptance, varied with isolation from butterflies in clearing populations, with the less isolated unlogged patches showing higher densities and greater *Collinsia* acceptance. The hypothesis that both these relationships were caused by dispersal out of clearings into the unlogged patches was supported when both relationships disappeared after all the clearing populations were temporarily extinguished by a June frost in 1992 (Singer, 2015; Singer & Thomas, 1996; Thomas et al., 1996).

Further evidence for dispersal‐driven maladaptation in unlogged patches at Rabbit Meadow comes from side‐by‐side preference comparisons performed in 1994, after the 1992 frost, between *Pedicularis*‐feeding butterflies in unlogged patches of the Rabbit Meadow metapopulation and *Pedicularis*‐feeding butterflies in the unlogged metapopulation at Colony Meadow that represented the starting condition for the Rabbit Meadow host shift. The preferences of *Pedicularis*‐feeding Rabbit Meadow butterflies were significantly more *Collinsia*‐accepting, showed a maladaptive legacy of immigration from clearings, after that immigration had ceased (Boughton, 1999; Singer, 2015; Singer & Thomas, 1996).

Given that *Pedicularis*‐preferring butterflies were biased to leave the clearings and were initially at high frequency there, we would expect a positive relationship between clearing patch size and butterfly density. Females in larger patches should be more likely to reach the oviposition motivation at which they would accept *Collinsia* before finding a patch edge and leaving. This expected relationship was found in 1986 across the Rabbit Meadow metapopulation. There was a threshold patch size below which most patches were not colonized; above that threshold larval density increased monotonically with patch size (Thomas et al., 1996). Experiments rejected the hypothesis that this effect was due to differences in acceptability or suitability between *Collinsias* in small and large patches. Instead, it was due to absence from the clearings of the host preferred by most butterflies.

**Figure 6:**
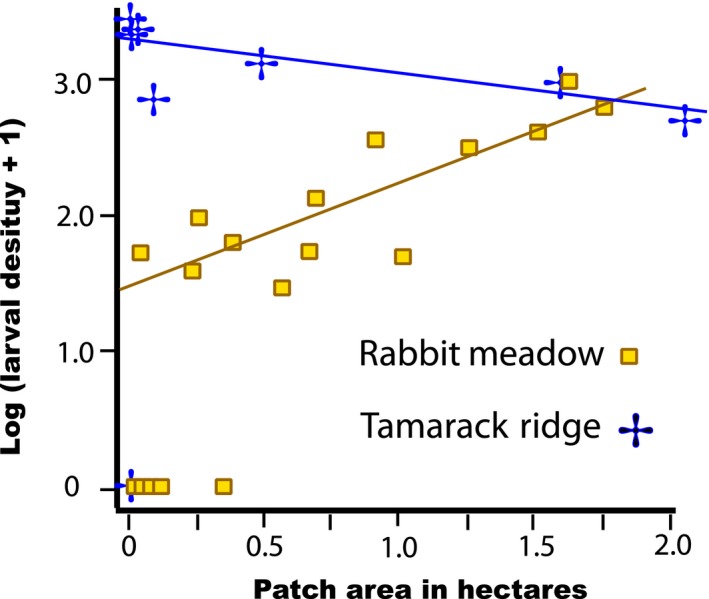
Relationships between LOG_10_ patch size of *Collinsia torreyi* and density of prediapause *E. editha* larval webs at Rabbit Meadow and at Tamarack Ridge (previously published, re‐drawn from Singer & Hanski, 2004; Rabbit data gathered by Chris Thomas, Tamarack data by MCS). Regression lines drawn using only occupied patches

Support for the hypothesis that the density/patch size relationship at Rabbit Meadow was caused by maladaptive emigration from clearings comes from comparison with Tamarack Ridge, where *Collinsia *was the preferred host and there was no trend for reduced density in smaller patches of it (Figure 6). Small patch size can engender maladaptation, as it does in crossbills (Siepielski & Benkman, 2005). However, in our system, we interpret the inability of the butterflies to colonize small patches at Rabbit Meadow as an effect of *pre‐existing* maladaptation to *Collinsia* on metapopulation dynamics, not as influence of patch size on adaptation.

#### 4.1.5 Fifth maladaptation: positive geotaxis exposes offspring to low‐quality food

**Figure 7:**
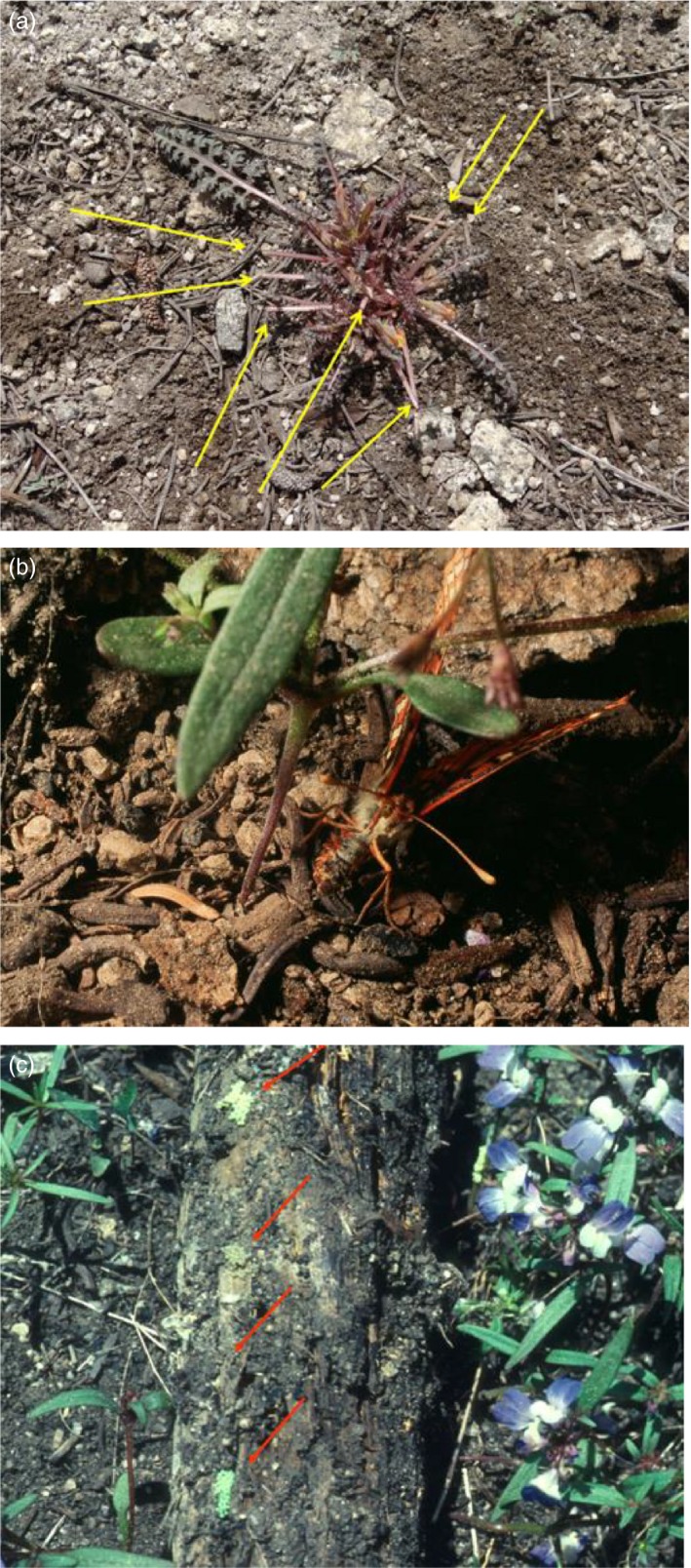
(a) A *Pedicularis semibarbata* plant at Rabbit Meadow on which the leaves that had been projecting highest have been naturally clipped by grazers. Grazed leaf stubs are indicated by arrows. Census results showing the proportion of *P. semibarbata* plants grazed like this are in Bennett et al. (2015). (b) An *E. editha* female at Rabbit Meadow that has accepted the chemical stimuli provided by *Collinsia torreyi*, has dropped to the ground and is searching with her ovipositor for the base of the plant, under which to tuck her abdomen and lay eggs. However, the plant has no base. (c) Four egg clutches of *E. editha* naturally laid under a log in a Rabbit Meadow clearing by butterflies responding positively to the taste of the (blue‐and‐white‐flowered) *Collinsia* plants surrounding the log. The log has been turned over by a human, to demonstrate the eggs

Grazing by vertebrates on *Pedicularis* (Figure 7a) has caused evolution of positive geotaxis by *Pedicularis*‐adapted *E. editha* (Bennett et al., 2015). This trait persisted in the clearing populations. In the manner of the positive geotaxis video linked above, a butterfly would respond to accepting the taste of *Collinsia* by dropping to the ground and searching for the base of the plant against which to press her ovipositor. However, *Collinsia torreyi* does not have a base. Figure 7b shows an *E. editha* female at Rabbit searching around a *Collinsia* with her tail for a nonexistent part of the plant. Many females were able eventually to oviposit on *Collinsia*, but for others their geotaxis expressed after accepting host chemistry resulted in failure to oviposit, oviposition on nearby nonhosts or eggs tucked under stones or logs. Figure 7c, a photograph taken in the Rabbit Meadow clearing, shows a log that had been turned over by a human searching for *E. editha* eggs. It bears four naturally laid clutches. *Euphydryas* eggs are adapted to being laid on transpiring leaves. If laid on logs they can die from desiccation in hot, dry weather. However, we observed that the majority of such clutches did survive, and that larvae from eggs laid on nonhosts could also survive if a host were within a few cm of the clutch.

Mean egg heights above the ground were 0.56 cm on *Pedicularis* at Rabbit and 4.8 & 5.1 cm in two *Collinsia*‐adapted metapopulations (Singer & McBride, 2010). When eggs were actually laid on *Collinsia* in a Rabbit clearing their mean height above the ground, measured in 1991, was 0.58 cm, so that the neonate larvae began feeding on the lower leaves of the plant. This, too, was maladaptive; when *Collinsias* were cut into basal, middle, and upper sections and fed to captive larvae, larval growth was faster on the tips than on central leaves and faster on the center than on the basal leaves (cotyledons) (McBride & Singer, 2010).

#### 4.1.6 Sixth maladaptation: clutch size on *Collinsia* larger than optimal

Experiments in which group sizes of neonate larvae were manipulated in the field showed that individuals survived significantly better in large groups on *Pedicularis* and in small groups on *Collinsia* (McBride & Singer, 2010). No surprise, then, that natural mean clutch sizes in the field ranged from 39 to 52 in four metapopulations adapted to *Pedicularis* and from 5 to 7 in three metapopulations adapted to *Collinsia* (Singer & McBride, 2010). Overall fecundity was not different; butterflies at Rabbit Meadow laid eggs once per day, but where clutches were small (e.g., Tamarack Ridge), oviposition was more frequent.

An adaptive response to colonizing *Collinsia *would be to reduce clutch size and increase oviposition frequency. In fact, mean clutch size at Rabbit Meadow in the field in 1982 was 50.9 (*n* = 50) on *Collinsia* and 43.5 (*n* = 79) on *Pedicularis*. The nonsignificant (*t* = 1.35, *df* = 127, *p* = 0.18) trend for clutches to be larger on *Collinsia* was in the opposite direction to that expected from adaptation to *Collinsia *(Singer, 2015). A similar trend was seen in insects captured newly eclosed in the clearing and offered only *Collinsia* or only *Pedicularis* for five days. For many of those butterflies, their first oviposition was delayed by a day or more on *Collinsia* compared to *Pedicularis,* and it was the most delayed insects that produced the largest clutches (Singer, 2015)*.*

Figure 5 of McBride and Singer (2010) indicates that experimentally increasing group size of neonate larvae from 5 to 30 reduced larval survival from 90% to 60% on blooming *Collinsia *and from 30% to 5% on senescent plants. Therefore, we expect a substantial negative impact on fitness of the Rabbit butterflies from the combination of their production of large clutches and preference for senescent plants.

## 5 EVOLUTION OF PREFERENCE 1980–1994

Because postalighting oviposition preferences of *E. editha* were heritable (McBride & Singer, 2010; Singer et al., 1988; Singer & Parmesan, 1993) and because variable preferences were actually expressed in nature at Rabbit Meadow, affecting the distribution of eggs across host genera (Singer, 1983), we expect those preferences to evolve during periods when diet is subject to natural selection. As expected, butterflies emerging from *Collinsia* in a Rabbit Meadow clearing, from eggs naturally laid on that host, were significantly less *Pedicularis*‐preferring in 1989 than in 1984 (Singer & Thomas, 1996). Preferences in unlogged patches changed in parallel, since the two patch types were sufficiently connected by dispersal (Section 4.1.4; Singer & Thomas, 1996). This evolution of increasing acceptance of *Collinsia* in the 1980s was reversed between 1989 and 1992 after *Collinsia*‐feeding butterflies in clearings across the metapopulation experienced a diversity of climate‐related catastrophes in three separate years: 1989, 1990, and 1992. Insects in the *Pedicularis*‐feeding patches across the metapopulation were immune to these disasters, and from 1989 to 1994, the metapopulation started to evolve away from postalighting acceptance of *Collinsia* (Singer & Thomas, 1996; Thomas et al., 1996).

## 6 TERMINAL PHASE, 2002–2003: MANIPULATED OVIPOSITION EXPERIMENTS SHOW BUTTERFLIES TUMBLED DOWN ADAPTIVE PEAK

The first year in which no natural oviposition was recorded on *Collinsia* across the entire Rabbit metapopulation was 2001. This observation implied that the evolutionary reduction of *Collinsia* acceptance recorded from 1989 to 1994 had continued. At the time, we wondered whether *Collinsia* may no longer support higher fitness than *Pedicularis* for Rabbit butterflies, and whether, if this were the case, the change in relative fitness on the two hosts might be ascribed to the butterflies’ continued maladaptations to their novel host.

To test this compound hypothesis, we estimated survival of both Rabbit Meadow and Tamarack Ridge larvae after manipulated ovipositions in cleared and unlogged patches at the Rabbit Meadow site. Rabbit Meadow butterflies were manipulated to oviposit on *Collinsia* in clearings and on *Pedicularis* in unlogged patches. Imported butterflies from Tamarack Ridge were tested only on *Collinsia*, since we had previously documented that they consistently reject *Pedicularis* (Singer & McBride, 2010). We give details of this experiment and its results here for the first time (prior reference to this result in Singer, Wee, Hawkins, & Butcher, 2008 was anecdotal, lacking experimental design, data, and analyses).

Each year we chose two patch‐pairs, with each pair comprising a clearing and an adjacent unlogged patch. One patch‐pair was used twice, in both years; the other two pairs were each used once, in a single year. Within each patch, we chose experimental plants by pacing out random numbers in a grid formation.

We captured female *E. editha* in the two metapopulations (Tamarack Ridge and Rabbit Meadow), fed them diluted honey, and kept them until they were just sufficiently oviposition‐motivated to accept at least some *Collinsias*. We then placed the butterflies upon the experimental plants. We were careful not to disturb the plants, since even small disturbances speed *Collinsia *senescence. When testing *Pedicularis* we used individual plants, but when testing the much smaller *Collinsias* we used natural clumps of plants growing within circles of about 3 cm diameter.

Each butterfly was allowed 5 min to decide whether to oviposit; if, after this time oviposition had not begun, we offered the butterfly a new plant. We deliberately staged these encounters with both blooming and senescent *Collinsia* plants in the clearings, choosing the two categories in alternation. By this means, we allowed the Rabbit Meadow butterflies to express their known oviposition preferences for senescent over blooming *Collinsia* and Tamarack Ridge butterflies to express their known preferences in the opposite direction (Singer & McBride, 2010).

Larvae of *E. editha* normally remain together as family groups through their first and second instars. After entering third instar, they quickly become more mobile, and by the middle of this instar, they are able to enter diapause if they cannot find food. Therefore, when following the fates of the experimental clutches, we recorded the numbers of larvae in each group that reached the beginning of third instar, at which time the experiment was terminated.

**Figure 8:**
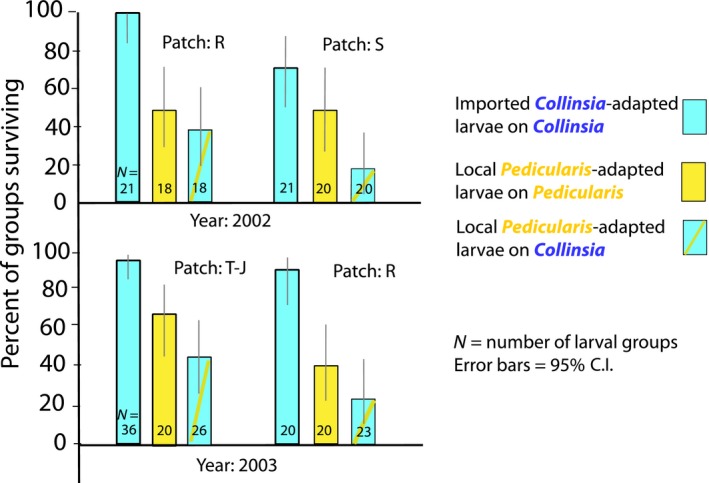
Group‐level survival (proportions of groups with surviving insects) through the egg stage and to the beginning of third instar in the manipulated oviposition experiment. All patches are within the Rabbit Meadow metapopulation (previously unpublished; raw data in Supporting information Table S1)

The four replicates of the manipulated oviposition experiment produced identical trends (Figure 8). The Figure shows proportions of larval groups surviving on each host in each patch, with error bars indicating 95% confidence limits calculated using “the confidence limit of a proportion” tab in vassarstats.net. The website gives references to the statistical literature that it uses. Raw data, including numbers of eggs laid and numbers of larvae surviving in each clutch, are in Supporting information Table S1.

Within each replicate, estimated survival was highest for the imported Tamarack Ridge (*Collinsia*‐adapted) insects on *Collinsia,* second highest for local Rabbit Meadow (*Pediculari*s‐adapted) insects on *Pedicularis* and lowest for local (Rabbit) insects on *Collinsia.* The principal cause of failure of local groups on *Collinsia* was senescence and death of the host, reflecting maladaptive host choice by the parents (Table 1). Estimates with overlapping confidence limits can differ significantly, but as it happens that was not the case for these data. Therefore, significance can be visualized from Figure 8, in which all comparisons with overlapping confidence limits are cases where differences did not reach significance at *p* < 0.05 (two‐tailed) within that replicate of the experiment. Conversely, nonoverlapping confidence limits do indicate significance.

**Table 1:** Role of host death in the survival and failure of Rabbit Meadow larval groups in the 2002–2003 manipulated oviposition experiment

Host	Groups surviving	Groups failing	Host dead	Host gone
*Collinsia*	27	57	51	3
*Pedicularis*	38	37	0	2

Notes

This is the same experiment depicted in Figure 8. Columns 2–3: absolute numbers of larval groups that survived or failed after oviposition on the two hosts. Columns 4–5: observed causes of failure: hosts died or disappeared. Where larvae are recorded as surviving, hosts were not dead. (All data previously unpublished.)

Figure 8 shows that, within three of the four replicates, imported *Collinsia*‐adapted larvae had significantly higher survival on *Collinsia* than did local *Pedicularis*‐adapted larvae on *Pedicularis*. However, the consistent trend for local Rabbit Meadow groups to survive better on *Pedicularis* than on *Collinsia* did not reach significance within any replicate. This trend was tested further with 3‐way log‐linear analyses of each year's data, to include patch identity (the three patches shown in Figure 8: R, S, and T‐J) and host effects for Rabbit Meadow insects on the two hosts but excluding the imported insects. Host effects were not significant within either year (*p* = 0.06 in 2002 and *p* = 0.09 in 2003). Neither did effects of patch identity reach significance in either year (*p* = 0.19 in 2002 and 0.06 in 2003).

If we simplify analysis by lumping the data from the four replicates of the experiment, then group‐level survival of Rabbit insects becomes significantly higher on their traditional host, *Pedicularis *than on *Collinsia,* as shown in Figure 8 (*p* = 0.024 by Fisher's exact test, two‐tailed; Table 1).

It would have been very useful to include in the experiment Rabbit Meadow butterflies emerging from *Collinsia,* whose changes of host preference we had studied through the 1980s (Singer & Thomas, 1996). Alas, this was no longer possible, as the clearing populations were extinct. We cannot exclude the possibility that *Collinsia*‐emerging butterflies, if they had still existed in 2002, would have been less enamored of senescent hosts than the *Pedicularis‐*emerging females that we tested. However, censuses of natural egg distributions up to the time of extinction of the *Collinsia‐*feeding populations suggest that preferences for *Collinsia* phenology did not change over this 14‐year period.

Combining our experimental results with long‐term censuses of eggs and larval webs in the field indicates that natural selection on host preference in 2002–2003 was toward use of the traditional host, *Pedicularis*, reversed from its direction at the beginning of our work in the 1980s when natural selection had favored oviposition on the novel host, *Collinsia*.

**Figure 9:**
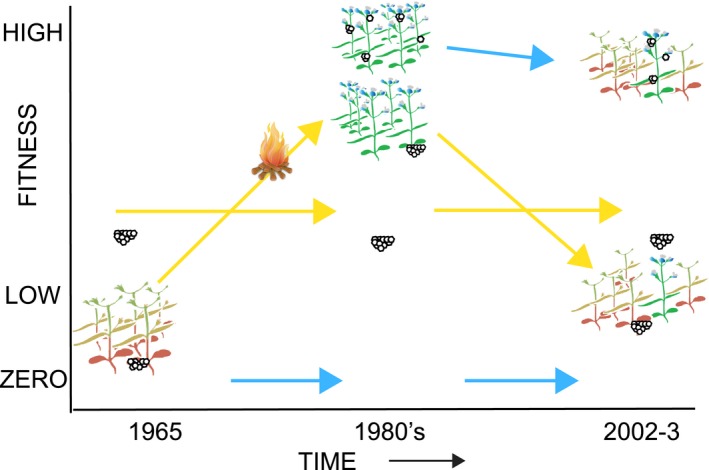
Stylized depiction of fitness changes across decades on both hosts at Rabbit Meadow for local *Pedicularis*‐adapted *E. editha* (yellow arrows) and imported *Collinsia*‐adapted *E. editha* from Tamarack Ridge (blue arrows). Blooming *Collinsia* are shown in green and blue, senescent are shown in red and orange. Eggs are shown as large clutches (30–50 eggs) naturally laid near ground level by *Pedicularis*‐adapted females and as small clutches (1–8 eggs) naturally laid higher up by *Collinsia*‐adapted females. Cartoon shows the bias toward young plants by *Collinsia*‐adapted females (small egg clutches on green/blue plants), and toward senescent plants by *Pedicularis*‐adapted females (large egg clutches on orange/red plants). Fitness of *Collinsia*‐adapted females on *Pedicularis* is shown along the “zero” line, because it is both unacceptable to ovipositing adults and unsuitable for larvae (Singer & McBride, 2010). Fitness of *Pedicularis*‐adapted females on *Collinsia *varied through time, approaching zero in the 1960s because of short host lifespan, climbing above fitness on *Pedicularis* after *Collinsia* lifespan was extended by logging, and dropping back after succession, but not quite as low as in the 1960s because, as depicted, *Collinsia* phenology was still more diverse than prelogging. While fitness on *Collinsia* was highest just after logging for females from both populations, that of *Pedicularis*‐adapted insects would never quite have reached the height of those adapted to *Collinsia* (McBride & Singer, 2010). We give no fitness estimate for Tamarack insects on *Collinsia* in the 1960s, since we do not know what proportion of plants would have lived long enough to support larvae to diapause. Fitness estimates in 2002–2003 of Tamarack insects on *Collinsia* and of Rabbit Meadow insects on both hosts are from the experiment shown in Figure 8

The dynamics over time of changes of fitness on the two hosts at Rabbit Meadow are summarized in stylized fashion in Figure 9. At the left is the starting condition, prior to logging, as judged from the unlogged patches in the 1980s (Moore, 1989), with very occasional oviposition by Rabbit insects on *Collinsias* that were likely to be senescent. In the center is the early stage of the host shift, with increased fitness on anthropogenically improved *Collinsia *and no change on *Pedicularis*. On the right is the result of the 2002–2003 experiment shown in Figure 8. Tamarack Ridge butterflies are shown in the two later time periods but not in 1965, because we know that they can have high fitness even when suitable *Collinsias* are infrequent (<1%), but we do not know what that frequency was in 1965. Positions and sizes of egg clutches on blooming and senescent *Collinsias* show the behavior of butterflies from the two origins.

## 7 THE SCHNEIDER HOST SHIFT: A SECOND CASE OF HIGHER FITNESS ON A NOVEL HOST TO WHICH BUTTERFLIES WERE NOT ADAPTED

Lest it seem an exceptional oddity that insects could immediately increase fitness by host‐shifting to a plant to which they were maladapted, we here summarize an independent but parallel event, also in *E. editha*. Butterflies at Schneider's Meadow, Carson City, Nevada, achieved an instant fitness gain by host‐shifting to a novel host to which they were not adapted. We describe this event briefly, since an up‐to‐date account has been published (Singer & Parmesan, 2018). In one sense, the host shift at Schneider's Meadow was the opposite of that at Rabbit Meadow. The Rabbit Meadow shift was from a persistent perennial to an ephemeral annual host; the Schneider's Meadow shift was in the opposite direction. In both cases, the starting condition was that no females preferred to oviposit on the novel host, but a small proportion accepted it readily (Singer & Thomas, 1996; Thomas et al., 1987). In both cases, there was no requirement of evolution for offspring survival to be higher on the novel host; this was the case from the first time that host was used (Singer & Parmesan, 2018; Singer & Thomas, 1996; Thomas et al., 1987). In both cases, our observations began in an early stage of the host shift when the novel host supported higher fitness than the traditional host, but was preferred by a minority of females. In both cases, postalighting preference for the novel host evolved and increased rapidly. In both cases, diet evolution was eventually returned to its starting point after environmental change exterminated insects on the novel host.

The traditional host at Schneider's Meadow was the short‐lived *Collinsia parviflora*, use of which resulted in the typical time constraint and fecundity/mortality trade‐off with consequent high mortality of prediapause larvae (Singer & Parmesan, 2018). After humans introduced a European weed, *Plantago lanceolata, *with a much longer lifespan than the *Collinsia, *host‐switching to *Plantago* instantly released the butterflies from their trade‐off, so that they achieved higher fitness on their novel host despite c.17% slower development on it (Singer & Parmesan, 2018).

The starting point of this episode of anthropogenic evolution was available for study in populations that had not colonized the exotic (Thomas et al., 1987), so we could show that individuals that laid eggs on the novel host achieved increased offspring survival from the moment that their population encountered that host. We could also show that no evolution of preference was necessary for colonizing *Plantago,* since 20% of adults in the ancestral condition accepted it readily, although none preferred it.

The butterflies would have been able to colonize *Plantago* as an ecological phenomenon, with no evolutionary change in either preference or performance. Such nonevolutionary shifts to novel habitats or resources are classified as examples of “ecological fitting” (Agosta, 2006; Araujo et al., 2015; Nylin et al., 2018). In general, host shifts and expansions of diet breadth in herbivorous insects occur with high frequency (Jahner, Bonilla, Badik, Shapiro, & Forister, 2011; Nylin et al., 2018; Strong, 1974) and seem not to be strongly constrained by trade‐offs (Forister & Jenkins, 2017; Gompert et al., 2015; Singer, Ng, Vasco, & Thomas, 1992).

In contrast to the host shift at Rabbit Meadow, in which we described the butterflies as “maladapted” to their novel host, we described the shift at Schneider's Meadow as driven by high fitness on a novel host to which the butterflies were “not adapted.” We hesitate to invoke “maladaptation” from the slower developmental rate on *Plantago* than on *Collinsia,* for we would wish to know that faster development on *Plantago* could have been achieved, and such an experiment was not done. Since the novel host was a recently introduced exotic, we could not compare the performance *E. editha* on that host with the performance of conspecific butterfly populations long‐adapted to that same host, as we did in the comparisons between Rabbit Meadow and Tamarack Ridge.

No matter how we choose to describe it in terms of adaptation, the high fitness on *Plantago* resulted in a complete host switch with rapid evolution of monophagy on the novel host, to the extent that the butterfly population was completely dependent on it by 2005. In 2008, a change of human land management rendered *Plantago *suddenly inaccessible, while leaving available the *Collinsia* that the insects had abandoned. The butterfly population suffered extinction, and then, after several years, the site was recolonized by butterflies monophagous on *Collinsia, *returning diet evolution to its starting point (Singer & Parmesan, 2018).

## 8 ADAPTATION MASQUERADING AS MALADAPTATION, AND VICE VERSA

Diverse, pervasive anthropogenic environmental change “should render many populations maladapted, leading to decreased individual fitness” (Hendry, Gotanda, & Svensson, 2017). There is much evidence for this (Burgess et al., 2018; Demeyrier, Lambrechts, Perret, & Gregoire, 2017; Fahrig, 2007; Frank et al., 2017; Keeler & Chew, 2008; Rogalski, 2017; Tillotson, Barnett, Bhuthimethee, Koehler & Quinn, 2019; Yoon & Read, 2016), although this special issue documents an unexpected exception in which polluted roadside environments increased frog fitness (Brady, Zamora‐Camacho et al., 2019).

As a cautionary tale in the context of these studies of anthropogenic effects, our study shows how appearances of both maladaptation and adaptation can be deceptive. First, routinely high mortality from phenological asynchrony with ephemeral hosts resembles a maladaptive outcome of climate change (Both, van Asch, Bijlsma, Berg, & Visser, 2009), but here it is neither an outcome of change nor maladaptive: It is adaptation masquerading as maladaptation (Singer & Parmesan, 2010). Second, when novel environments immediately support increased fitness the appearance that change has strengthened adaptation can be equally false: fitness gain can disguise maladaptation to a novel environment. Both categories of paradox are exemplified by our study insects.

## 9 CONCLUSION

The set of apparent paradoxes listed in our opening paragraph all applied to the Rabbit Meadow metapopulation in the 1980s:

1. Fitness was higher on the host to which the butterflies were maladapted.
2. Habitats to which the insects were maladapted acted as sources and the patches to which they were adapted acted as apparent sinks (pseudosinks).
3. By dispersing out of the habitat patches to which they were maladapted and into the habitats to which they were adapted most females reduced not only their own fitnesses but the fitnesses of the populations into which they immigrated.

In *E. editha* populations that were not undergoing rapid diet evolution, we found a general concordance between rank orders of plants in the oviposition preference hierarchy and in the ability of those plants to support offspring survival (Figure 1). This concordance in a set of populations with diverse diets suggests that, in the medium term, adaptation to different host genera within the species’ current host range is not constrained. Therefore, both the Rabbit Meadow and Schneider's Meadow insects would likely have eventually evolved local adaptation to their novel hosts, had they not been derailed by rapid environmental change.

However, within the timeframe of our study, the only host‐adaptive trait that clearly evolved at Rabbit Meadow was postalighting preference (Singer & Thomas, 1996). We did not apply repeated quantitative assessments to other traits, but, over the duration of our study, we observed that the butterflies continued to find *Collinsia *inefficiently, to prefer senescent over blooming individuals, and to lay maladaptively large clutches at the bases of the plants. The manipulated oviposition experiment suggests that one of these maladaptations was crucial to the demise of the insects on their novel host.

As succession proceeded and *Collinsia* declined in quality, the preference of the butterflies for senescent plants assumed greater importance, until the fitness advantage on the novel host was reversed. Lack of adaptation to *Collinsia* in this specific trait allowed successional change to increase larval mortality caused by host death from 12% in 1984–1986 (Moore, 1989) to 61% in 2002–2003 (Table 1). This change reversed the direction of natural selection on diet to favor oviposition on *Pedicularis* and helps explain the abandonment of *Collinsia* at a time when, as the manipulated oviposition experiments showed, this host would still have provided the highest fitness in the habitat, given appropriate adaptation.

In contrast, the extinction at Schneider's Meadow was not due to lack of adaptation to the novel host *Plantago*, but to the occurrence of an unprecedented type of anthropogenic environmental change, at a pace that could not be matched by insect evolution. Better adaptation to *Plantago* would not have helped, as witnessed by widespread population extinctions of butterflies adapted to the same *Plantago* species (*P. lanceolata)* in Europe, in response to abandonment of traditional grazing and haymaking (Wallis de Vries & van Swaay, 2006). Butterflies can and do evolve rapidly, but humans can alter butterfly habitats even faster than butterflies can evolve (Singer & Parmesan, 2018).

Despite their different trajectories, the Rabbit Meadow and Schneider's Meadow histories both illustrate how host shifts can start out as purely ecological events. Populations can instantly achieve increased fitness on novel resources, without the need for evolutionary change and, in the case of the Rabbit shift, despite carrying a suite of clear maladaptations to the novel resource. Once the shifts had occurred, rapid evolution quickly followed in response to natural selection to prefer the novel over the traditional hosts that were still present.

These effects, cryptic without the level of detailed study presented here, are surely under‐appreciated forces in evolution and ecology. Yet understanding them is pertinent to asking whether conservation practices should strive to maximize adaptation or evolvability (Derry et al., 2019). Our studies of *E. editha* suggest that evolvability is more important to persistence of populations than are specific adaptations to particular resources, which can be ephemeral. For our system, it is clear that possession of adaptation to novel resources has not been necessary for short‐term adoption of those resources, but that evolutionary flexibility has been essential for persistence of populations after their host shifts have been achieved.

## 10 GLOSSARY

### 10.1 Pseudosink

A habitat patch that on balance receives more immigrants than it emits emigrants and thereby appears to be a sink. However, if immigration is cut off, a pseudosink does not decline to extinction, but stabilizes at a lower population density (Watkinson & Sutherland, 1995).

### 10.2 Host use

In this paper, the proportion of eggs laid on each host species by an insect population. The definition could equally be applied to distributions of larvae across hosts, but we here ignore that; Lepidopteran larvae often show lower host specialization than ovipositing adults when they undertake their independent host searches after they have developed sufficiently to do so (Wiklund, 1974).

### 10.3 Insect preference

The set of likelihoods of accepting particular specified hosts that are encountered. Defined in this way, it is a property of the insect that can vary among individuals (Singer, 2000) and can be heritable. As described in this paper E. editha first encounters hosts visually, then chemically, then physically, with separate preferences expressed at each stage. Again in E. editha, strength of postalighting preference for two hosts, say host A and host B, is measured by the length of time that a female will search accepting only host B (if encountered) until, after failing to find host B, she reaches the level of oviposition motivation at which either A or B would be accepted, whichever is next encountered (details and justification in Singer, Vasco et al., 1992).

### 10.4 Plant acceptability

The set of likelihoods that a plant will be accepted by particular specified insects that encounter it. Defined in this way, it is a mirror‐image of preference, a property of the plant that can vary among individuals (Singer, 2000) and can be heritable.

### 10.5 Plant apparency

The set of likelihoods that a plant will be perceived by particular specified insects that approach it (Singer, 2000).

### 10.6 Site and patch

In this paper, we use “oviposition site” to mean an exact point where eggs are laid, but we also use “site” without the “oviposition” prefix to indicate a much larger area, a habitat occupied by a discrete population or metapopulation of butterflies. A “patch” is an area within a metapopulation capable of harboring a population that could exchange individuals with other patches in the same metapopulation.

## 11 DATA ARCHIVING STATEMENT

Data from the experiment shown in Figures 8 and 9 are available as Supporting information in Table S1.

## CONFLICT OF INTEREST

The authors declare no conflict of interest or competing financial interests.
